# Coral-like BaTiO_3_-Filled Polymeric Composites as Piezoelectric Nanogenerators for Movement Sensing

**DOI:** 10.3390/polym15153191

**Published:** 2023-07-27

**Authors:** Yuhang Du, Gang Jian, Chen Zhang, Fengwei Wang

**Affiliations:** 1 School of Materials Science and Engineering, Jiangsu University of Science and Technology, Zhenjiang 212100, China; dyh211@163.com (Y.D.); czhang1981@hotmail.com (C.Z.); fwwang@just.edu.cn (F.W.); 2 Shenzhen Institute of Advanced Electronic Materials, Shenzhen Institutes of Advanced Technology, Chinese Academy of Sciences, Shenzhen 518055, China

**Keywords:** piezoelectric energy harvesting, three-dimensional coral-like BaTiO_3_, polyvinylidene fluoride (PVDF), flexible

## Abstract

Piezoelectric nanogenerators have prospective uses for generating mechanical energy and powering electronic devices due to their high output and flexible behavior. In this research, the synthesis of the three-dimensional coral-like BaTiO_3_ (CBT) and its filling into a polyvinylidene fluoride (PVDF) matrix to obtain composites with excellent energy harvesting properties are reported. The CBT-based PENG has a 163 V voltage and a 16.7 µA current at a frequency of 4 Hz with 50 N compression. Simulations show that the high local stresses in the CBT coral branch structure are the main reason for the improved performance. The piezoelectric nanogenerator showed good durability at 5000 cycles, and 50 commercial light-emitting diodes were turned on. The piezoelectric nanogenerator generates a voltage of 4.68–12 V to capture the energy generated by the ball falling from different heights and a voltage of ≈0.55 V to capture the mechanical energy of the ball’s movement as it passes. This study suggests a CBT-based piezoelectric nanogenerator for potential use in piezoelectric sensors that has dramatically improved energy harvesting characteristics.

## 1. Introduction

The development of portable and wearable electronic devices is a newly emerging field of science and technology that has been extensively researched in recent years and has been widely used in devices required for human life, such as electronic communication devices and health monitoring sensors [1,2]. Despite the convenience offered by these technologies, the conventional power supply used in the above-mentioned devices is still based on energy conversion devices (lithium batteries, lead-acid batteries, etc.) made of piezoelectric crystals and ceramics, represented by the quartz and acid-lead series [3]. Conventional batteries have limited capacity, and some potential problems are beginning to emerge. In addition, the general shortage of conventional energy sources is increasing the demand for sustainable, self-powered devices that can draw energy directly from the various energy sources present in the environment (e.g., solar energy, thermal gradients, and mechanical vibrations) [4,5,6,7,8,9,10]. Recently, piezoelectric nanogenerators (PENGs) have been used to generate electrical energy from one of the most abundant sources of energy in our environment (mechanical energy) [11]. By combining vitamin B2 (VB2) with PVDF, Karan et al. have designed an entirely organic-based, biocompatible piezoelectric nanogenerator (PENG) that can harvest green, clean electricity from air flowing as wind energy or raindrops falling as blue energy [12]. Zhang et al. designed a self-powered pacemaker based on all-in-one piezoelectric nanogenerator (A-PENG), a device that derives biomechanical energy from the beating heart [13]. Jiao et al. have developed a novel nanogenerator (MM-PENG) based on hexagonally corrugated plate-like mechanical metamaterials (MM) that efficiently generates electricity under quasi-static environmental excitation [14]. The composite PENG based on inorganic piezoelectric materials and organic polymers is a promising self-powered generator with good flexibility and high-voltage electrical performance [15,16].

Barium titanate is an important inorganic piezoelectric material that has a good piezoelectric effect and is therefore widely used in the manufacture of electronic components such as piezoelectric sensors, piezoelectric ceramic capacitors, and piezoelectric oscillators. However, due to the rigidity and brittleness of barium titanate ceramics, they are not well suited to the requirements of flexible and wearable electronic product design. Polyvinylidene fluoride (PVDF) is a flexible, stretchable organic polymer piezoelectric material [17,18]. Although PVDF is flexible, it is not widely used because of its low piezoelectric properties [19]. Thus, piezoelectric polymer composites were designed to combine the advantages of the high voltage electrical coefficient of the embedded ceramic filler with the mechanical flexibility and ease of processing of the polymer collective for flexible energy harvesting devices [20,21,22]. Additionally, the piezoelectric filler stimulates PVDF crystallization and also has a separate piezoelectric effect [23,24,25]. Currently, apart from BaTiO_3_(BT), ZnO, AIN, and Pb(Zr, Ti)O_3_(PZT) can be used as piezoelectric materials in TENG, and both BT and AIN are environmentally friendly and relatively biocompatible. Although PZT can exhibit good piezoelectric properties, its pollution of the environment is currently a major problem [26,27,28]. By using a non-solvent-induced phase separation technique, Tian et al. created a flexible piezoelectric sensor based on PZT nanoparticles embedded in a PVDF polymer matrix [29]. By etching ZnO nanoparticles in ZnO/PVDF nanocomposites with hydrochloric acid (HCl) and casting them on a flat surface, Mao et al. created a mesoporous piezoelectric PVDF film [30]. Jiang et al. used an AIN-doped P(VDF-TrFE) polymer matrix to create a flexible wearable piezoelectric nanosensor [31].

A piezoelectric composite is a combination of two piezoelectric materials, and the piezoelectric properties of the resulting material can be improved by combining them in different ways [32]. The preparation methods of piezoelectric film materials include vacuum evaporation coating, sputtering coating, chemical vapor deposition coating, the new sol-gel method, hydrothermal methods, electrophoretic deposition methods, etc. The future preparation technology will develop in the direction of high efficiency, low cost, and high quality [33]. There is no doubt that composites can advance piezoelectric technology. In piezoelectric polymeric composites, the external mechanical forces applied are actually difficult to reach the filler, and the polymer collectively consumes and absorbs most of the mechanical forces. Inefficient load transfer due to large differences in the stiffness of the material composition and the spatial discontinuity of the ceramic phase [19]. The increased continuity of the filler is feasible in terms of significantly improving the performance of the PENG [4,34]. In addition, the shape of the filler also plays a role in the piezoelectric properties of the composite [34,35,36,37,38,39].

In this work, preliminary coral-like BT (CBT)-filled PVDF composites were prepared, and PENG with significant enhancement properties is reported. Briefly, 163 V and 16.7 μA are the voltage and current values for a PENG based on a single layer CBT. The electrical energy of PENG can light up commercial light-emitting diodes. High local stresses were observed in the CBT dendritic structure through finite element simulations, which suggests that the unique filler morphology plays a crucial role in achieving high performance. In addition, the PENG is able to collect the energy of falling balls at different heights and the mechanical energy of the ball’s movement through it by means of a high voltage signal. Therefore, the PENG suggested in this study is a good option for the conversion of various mechanical energies into electrical energy.

## 2. Materials and Methods

### 2.1. Preparation of the Coral-like BaTiO_3_

Synthesis of coral-like BT nanoparticles by the hydrothermal method All reagents are of analytical grade purity. Briefly, 35 mL of deionized water was heated to boiling to remove soluble carbon dioxide, and 0.1 g of hexadecyltrimethylammonium bromide (99%, Sigma-Aladdin, Shanghai, China) was then added to the pre-treated deionized water. After 10 min of magnetic stirring, a homogeneous solution is obtained. Then, 2 g of Ba(OH)_2_-8H_2_O (99.5%, Sigma-Aladdin, Shanghai, China) was dissolved in the above solution, and 2 mL of tetrabutyl titanate (99%, Sigma-Aladdin, Shanghai, China) was mixed with the solution by vigorous stirring for 10 min and transferred to a 50 mL reaction vessel. The reaction kettle was heated in an oven at 180 °C for 12 h. The resulting product was washed several times, each time with ethanol and distilled water. The final product was dried overnight at 50 °C under vacuum for further characterization.

### 2.2. Preparation of the PVDF/Coral-like BaTiO_3_ Film and PENG

Briefly, 10 g of N, N-dimethylformamide (99.8%, Sigma-Aladdin, Shanghai, China) solution was mixed with 1 g of PVDF powder (761A, Arkema, Paris, France). The mixture was stirred for 36 h to completely dissolve the polymer into a clear solution, and then the synthetic coral-like BT particles were dispersed in the above mixture and stirred for 5–6 h to have a uniform dispersion. The solution was poured with a spatula onto a flat glass of controlled area and thickness. The solution was left at 80 °C for 3 h to remove the solution, then heat-treated to 120 °C and kept for 1 h to obtain the composite. All samples of PVDF and nanocomposite films were ~7 μm thick and cut to 2 cm × 4 cm for the preparation of PENG. Both surfaces of the composite were covered with aluminum tape (425-1, 3M, Maplewood, MN, USA), which serves as an electrode, and sealed overall with polyimide tape (PI, 5413D, 3M, Maplewood, MN, USA). Prior to electrical measurement, the manufactured PENG was polarized at 2 kV for 24 h at room temperature.

### 2.3. Characterization and Electric Measurement

SEM measurements were performed with Hitachi S4800 (Tokyo, Japan) and JEOL JSM6480 (Tokyo, Japan). XRD measurements were carried out on a PANalytical B.V. diffractometer (Malvern, UK). Compressive stress-strain measurements were carried out using TA Q800 Dynamic mechanical analysis equipment (TA Instruments, New Castle, DE, USA) at strain rates ranging from 10^−2^ s^−1^ to 25% strain. The samples were formed into cylindrical shapes with a diameter of 5 mm and a thickness of 1 mm for mechanical tests. Using a Tektronix TBS1072B (Beaverton, OR, USA) data storage oscilloscope with a 100 MΩ internal resistance, the open-circuit voltage of the PENG was measured. A Keithley 6514 (Cleveland, OH, USA) electrostatic meter was used to measure the short circuit current. The dynamic pressure applied to the PENG is measured using a digital force meter (EP Metrology Instruments, Shenzhen, China).

### 2.4. Simulation

Finite element analysis was performed in 2D mode on COMSOL 6.0 with a model size of 5 µm × 5 µm. In the constructed model, the CBT was randomly distributed in the PVDF matrix. The forces applied to the top and bottom edges were 50 N and 0 N, respectively. Appropriate dielectric constants, piezoelectric coupling matrices, and elasticity matrices for components (BT and PVDF) were added to the simulated system. The solution for the potential and stress distribution in the PVDF composite PENG was obtained by analysis.

## 3. Results

**Figure 1:**
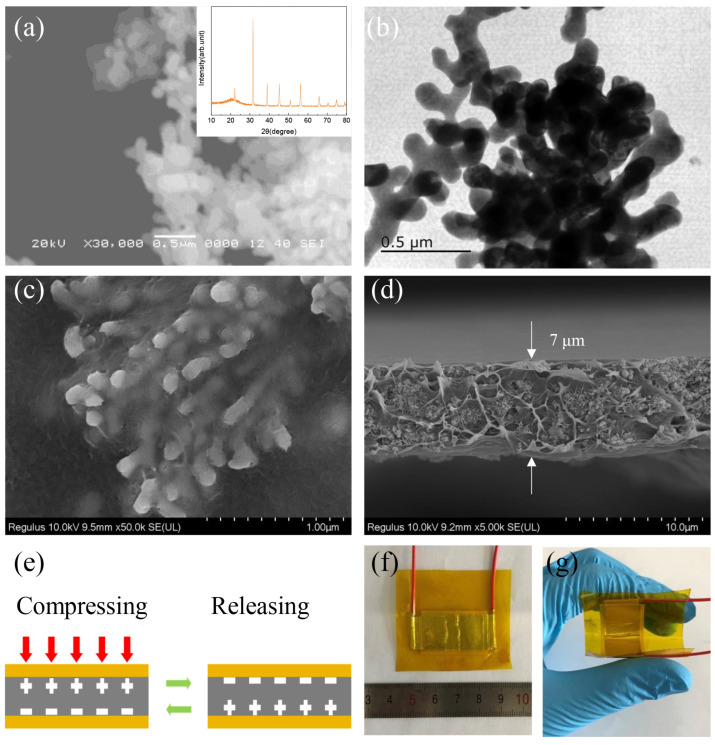
(**a**) SEM image and XRD pattern of coral-like BT particles. (**b**) TEM image of coral-like BT. (**c**,**d**) SEM image of coral-like BT/PVDF composites. Coral-like BT/PVDF. (**e**) Diagram depicting the PENG in this study’s operating modes. (**f**,**g**) Photographs of the coral-like BT/PVDF energy harvester.

The pre-synthesized coral-like BT is depicted in Figure 1a as a scanning electron microscopy (SEM) picture. The prepared particles are arranged freely in a dendritic pattern, showing the morphology of coral branches. The coral branches do not break down even after prolonged sonication, indicating that the synthetic CBT has a solid three-dimensional morphology. The inset in Figure 1a displays X-ray diffraction (XRD) plots of the manufactured coral-like particles. The particles are well crystalline and belong to the cubic BT phase, JCPDS No. 89-2475 [40]. Figure 1b shows the morphology of the CBT under TEM. SEM images of the CBT-PVDF composite are shown in Figure 1c,d. From these images, a dense film with a thickness of roughly 7 μm and a uniform distribution of morphologies can be seen. Figure 1e shows the mode of operation of the PENG in this research. When polarized at high voltage, the electric dipole moments (i.e., the distance between the centers of positive and negative ions) in the material are aligned in a directional manner. Positive piezoelectric voltage is produced in compression mode as the electric dipole moment is stretched under external force. A negative voltage is produced in the release mode as a result of the material’s inverse elastic effect, which shortens the length of the electric dipole moment. Images of the PENG of the constructed CBT-PVDF composite are presented in Figure 1f,g, where it is possible to see the PENG’s excellent flexibility. It is noteworthy that even after many bends, the PENG keeps its robust structure. The PENG is very adaptable to the application and has a flexible design that allows it to be fixed in locations that are not completely flat.

**Figure 2:**
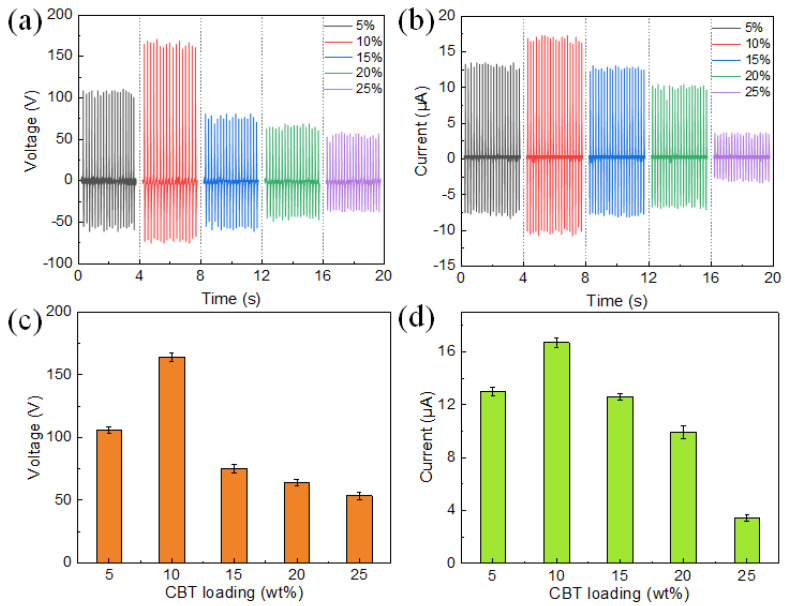
(**a**) The coral-like BT/PVDF PENG’s open-circuit voltage and (**b**) short-circuit current were measured with different coral-like BT loadings at a compressive force of 50 N and a frequency of 3.5 Hz. (**c**) At a compressive force of 50 N and a frequency of 3.5 Hz, the peak open-circuit voltage and (**d**) peak short-circuit current of the coral-like BT/PVDF are shown as functions of coral-like BT loadings.

Figure 2a,b display the output of the open circuit voltage (V_oc_) and short circuit current (I_sc_) of a CBT-PVDF PENG (approximately 7 μm thick, 2 cm × 4 cm) at 3.5 Hz, 50 N compression, with different CBT fillings. The PENG produces high voltages of up to several hundred volts and currents of tens of microamps, demonstrating its excellent performance. In addition, both voltage and current show a high dependence on CBT filling. Voltage and current first rise and then fall as the CBT rises from 5% to 25%, reaching their peak at 10%. This rise results from the improved piezoelectricity as the amount of CBT filling in the composite increases. A dipole exists intrinsically in the BT lattice, making it a high-voltage electrical material [41]. The piezoelectric principle of BT is based on the special properties of its crystal structure. The crystal structure of BT is of the chalcogenide type, with the titanium and oxygen ions in its lattice showing an asymmetrical arrangement, forming an electric dipole. When an external force is applied, the lattice structure is distorted, resulting in a change in the direction of the electric dipole, which generates an electric charge. The decrease at 15%–25% CBT may be brought on by the film structure degrading at high filler loading. Figure 2c,d illustrate the positive peak voltage and current of the PENG at various CBT loads (i.e., the positive maximum to zero). At CBT filler loadings of 5%, 10%, 15%, 20%, and 25%, the PENG obtained V_oc_ of 105 V, 163 V, 75 V, 64 V, and 53 V; I_sc_ of 12.9 μA, 16.7 μA, 12.6 μA, 9.9 μA, and 3.4 μA, respectively.

**Figure 3:**
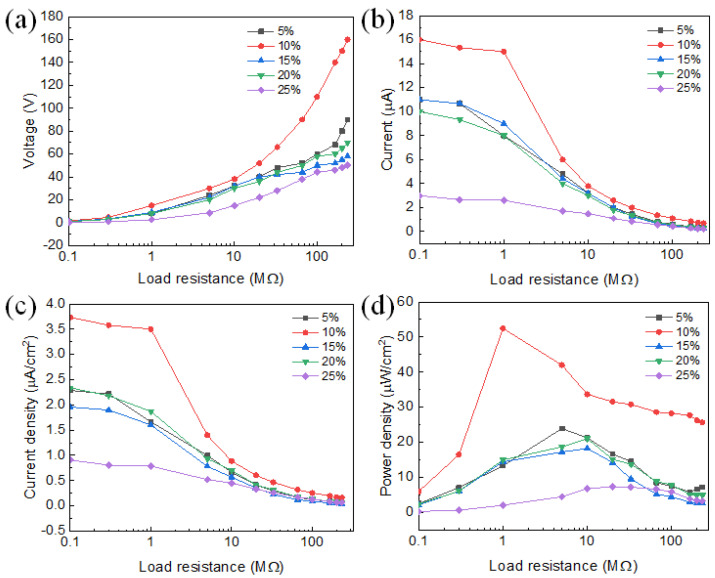
Coral-like BT/PVDF PENGs that resemble coral are shown in (**a**) the open-circuit voltage, (**b**) the current, (**c**) the current density, and (**d**) the power density as functions of load resistance at a compressive stress of 50 N and at a frequency of 3.5 Hz.

Under various external load resistances, the voltage, current, and power output of the CBT/PVDF PENG were studied. Figure 3a,b display the PENG’s peak open circuit voltage and current at various load resistances. As the load resistance increases, the voltage increases, and the current decreases. This is due to the fact that the load resistance increases, the current decreases, and the voltage division increases. The findings demonstrate that the 10% CBT/PVDF PENG has the highest voltage and current among the PENGs with various CBT loads, with voltage increasing from 4.6 V at 100 kΩ to 160 V at 233 MΩ and current decreasing from 16 μA at 100 kΩ to 1 μA at 233 MΩ. The peak current density of the PENG at various load resistances and CBT loads is shown in Figure 3c. The current density of the 10% CBT/PVDF PENG is 3.7 μA cm^−2^ and 0.16 μA cm^−2^ at 100 kΩ and 233 MΩ, respectively. Power is calculated from P = I^2^R, where I and R are the current and load resistance, respectively. A maximum value is attained with a maximum power density of 52.5 μWcm^−2^ in Figure 3d, where the power density is correlated with the external load resistance. The maximum power of CBT/PVDF PENG with CBT fillings of 5%, 15%, 20%, and 25% was 23.9 μWcm^−2^, 21 μWcm^−2^, 18.2 μWcm^−2^, and 7.3 μWcm^−2^, respectively.

**Figure 4:**
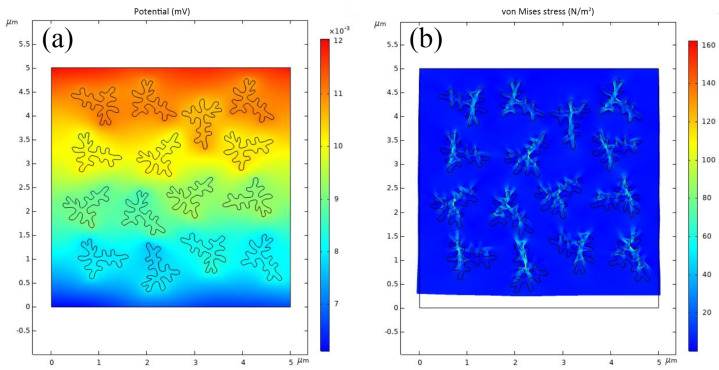
The simulated distribution of (**a**) electric potential and (**b**) stress in coral-like BT/PVDF PENG under a compression of 50 N at 3.5 Hz is shown using finite element analysis (FEA).

In order to determine the mechanism behind the enhanced performance of the CBT/PVDF-based PENG, a finite element analysis of the PENG was conducted. Figure 4a,b show the simulated potential and stresses of the CBT/PVDF composite under 50 N compression. As can be seen in Figure 4b, the red part of the CBT is the part with high stress. The structure of the three-dimensional coral branch increases its surface area and surface energy, thus increasing its contact area and contact energy and making it more sensitive to the corresponding external pressure. Under the external pressure applied to the composite, the stresses are concentrated in the BT coral branches. When external pressure is applied to the branching structure of the three-dimensional coral branch, it produces deformations, such as bending and twisting, which result in local stress concentrations.

The effectiveness of PENGs in this study and previous published work is compared in Table 1. In this work, the PENG using CBT as a filler performed well compared to literature using various nano-fillers and polymeric substrates. Voltages of 2.6–220 V and currents of 22 nA–19 μA are depicted in these publications [18,37,39,42,43,44,45]. In this study, the associated CBT/PVDF parameters all exhibit high values. It is important to note that the CBT in this work has a unique morphology that allows it to increase continuity, and the composite has excellent flexibility, which helps it operate well.

**Figure 5:**
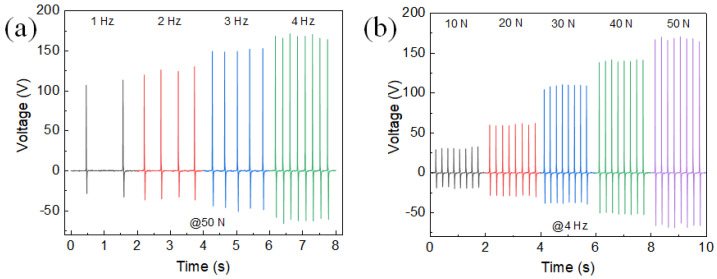
Performances of the 10% coral-like BT/PVDF PENG. (**a**) The 10% coral-like BT/PVDF PENG’s output voltage in open circuit at various frequencies. (**b**) The 10% coral-like BT/PVDF PENG’s open-circuit voltage outputs at varied compressive stresses.

Figure 5a shows the voltage at different frequencies for a 10% CBT/PVDF PENG at a fixed compression of 50 N. The output voltage of the PENG is frequency-dependent and is 113 V, 130 V, 153 V, and 171 V at 1 Hz, 2 Hz, 3 Hz, and 4 Hz, respectively. The frequency characteristics of the PENG show that relatively good performance can be achieved when the PENG is operated at higher frequencies. As the frequency increases, it leads to an increase in the frequency of vibration of the piezoelectric material, resulting in more charge being generated. At the same time, an increase in frequency also causes the amplitude of the material to vibrate less, but the increase in charge results in an increase in the total output voltage. Figure 5b shows the voltage output of a 10% CBT/PVDF PENG with varying compression forces at a fixed frequency of 4 Hz. The results show a linear relationship between the output voltage and the applied pressure when compressed from 10 N to 50 N, i.e., the output voltage increases linearly with increasing pressure. When a piezoelectric material is subjected to pressure, it undergoes distortion which results in an electrical charge. When a greater force is applied, the degree of distortion increases, as does the amount of charge generated, leading to an increase in output voltage.

**Figure 6:**
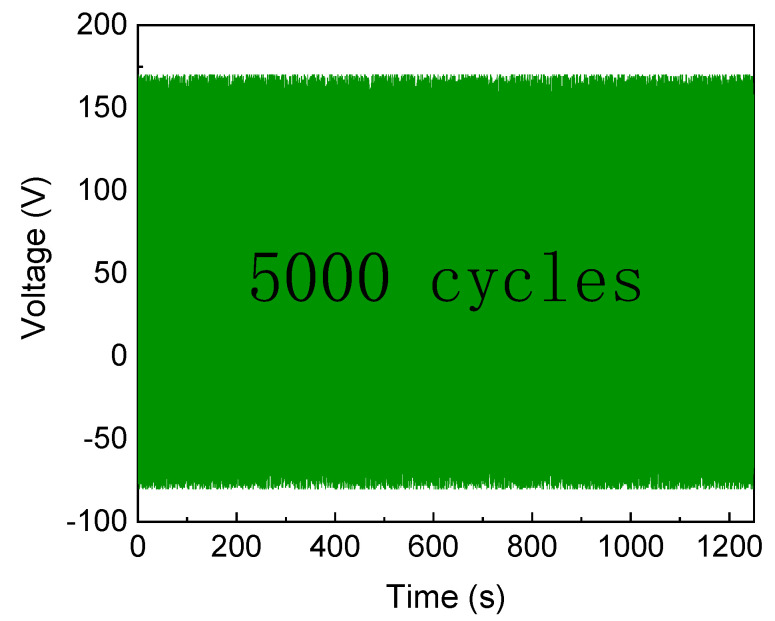
CBT/PVDF PENG open-circuit voltage output at 50 N compression force at 3.5 Hz for 5000 cycles.

Durability tests on the PENG were conducted to gauge its stability, and the findings are displayed in Figure 6. The durability test is carried out in an indoor environment at room temperature at 4 Hz under a cyclic pressure of 50 N. A layered device structure was used for the tests (PI/AI/PENG/AI/PI, with the AI and PI bands being the electrode and sealing material, respectively). The morphology of the PENG was well maintained after 5000 cycles of compressions and releases, showing that the PENG has good durability and stability. No substantial voltage degradation was noticed during this time.

**Figure 7:**
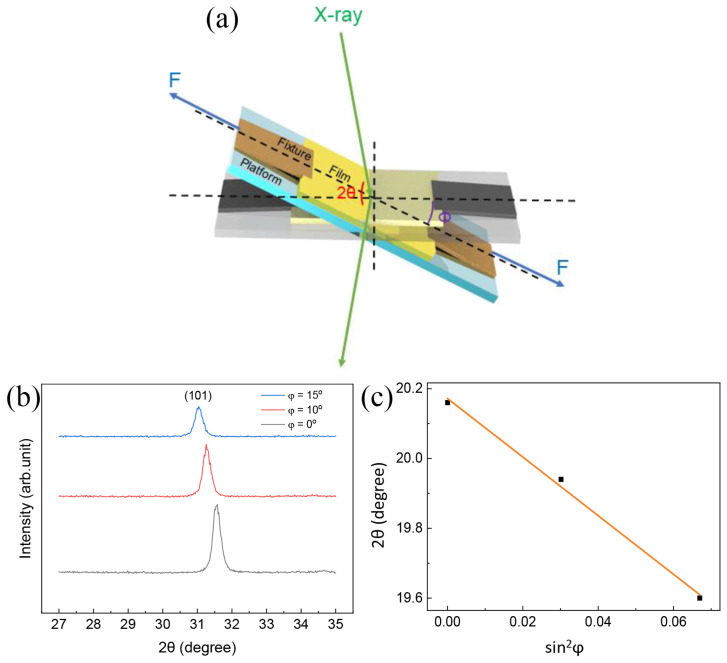
(**a**) Diagram showing how stress is measured by XRD in coral-like BT/PVDF composite. A steady stretching tension of 7.15 Mpa is applied to the sample. (**b**) Coral-like BT/PVDF composite XRD patterns at sample tilt angles of = 0°, 10°, and 15°, respectively. (**c**) The (101) diffraction peak for BT derived from XRD patterns, along with its fitted line of 2θ versus sin^2^*φ*.

Figure 7a illustrates the XRD pattern results of the sample at different tilt angles for examining the local stresses in the CBT/PVDF composite under external strain. During the measurement, a constant tensile stress of approximately 7.15 Mpa is applied to the sample. Calculating σ = M·K, where K is the elasticity constant and M is the slope of the 2θ-sin2φ plot in the XRD pattern, yields the residual stress. [46,47]:

(1) $$K=-\frac{E}{2\left(1+\nu \right)}\cdot \frac{\pi }{180{}^{\circ}}cot\theta {}^{\circ}$$

where *E* is the Young’s modulus, ν is the sample’s Poisson’s ratio, and *θ*° is the tilt angle of the research peak ([101] of BT) at tilt angle *φ* = 0. Figure 7b shows the residual stresses measured in the XRD pattern. It was discovered that tensile stress caused the composite’s diffraction peak to shift toward a smaller *θ* inclination as the inclination *φ* grew. Considering the highest diffraction peaks of BT of (101), 2θ is 20.16°, 19.94°, and 19.6° for *φ* = 0°, 10° and 15°, respectively. BT’s elastic characteristics of 135 Gpa and 0.32 for ν [48,49], There are significant local stresses in the BT coral, as shown by the computed stress of 17.08 Gpa for the CBT in the composite, which is substantially higher than the external stress of roughly 7.15 Mpa applied to the entire sample (Figure 7c). A great number of voids and branching in the coral-like structure have an effect on the distribution and transfer of stresses, causing local stresses to be concentrated in specific areas. It is inferred that high local stresses are also present in the BT coral when external compressive stresses are applied. The enhanced piezoelectric property is therefore due to the irradiated morphology of the BT coral dendrites in the composite.

**Figure 8:**
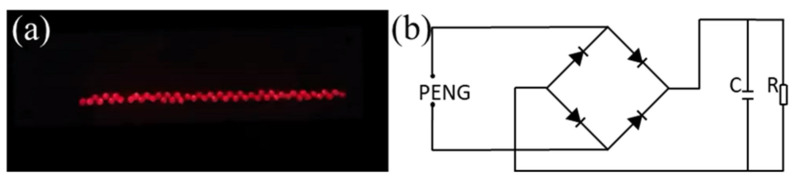
(**a**) Without using storage capacitors, 10% CBT/PVDF PENG directly illuminates 50 commercial LEDs. (**b**) Diagrammatic representation of the rectifier circuit.

There are several uses for the electrical energy that the PENG produces. Due to the high instantaneous voltage and current produced by the PENG, Figure 8a depicts 50 commercial light-emitting diodes (LEDs) being illuminated immediately by a 10% CBT/PVDF PENG without any charge storage (Video S1, Supplementary Materials). Other situations call for rectifying the current and storing it in power electronics, which need lower voltage amplitudes and higher currents [50,51]. A schematic of the full wave rectifier employed in this work is shown in Figure 8b.

**Figure 9:**
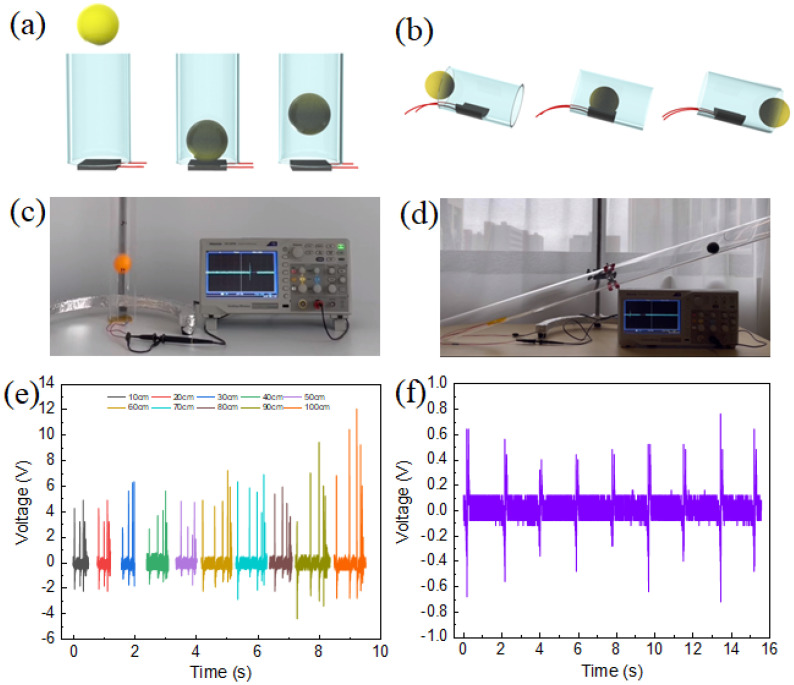
Demonstration of the application of the energy harvester. (**a**) Schematic diagram of energy harvesting for ball drop, (**b**) Schematic diagram of ball passage signal monitoring, (**c**,**e**) application of 10% CBT/PVDF PENG in ball-drop motion, and (**d**,**f**) 10% CBT/PVDF PENG in blob passing signal detection single application.

As an energy collector, the PENG can be used to capture all kinds of compressed energy, such as road energy, daily life energy, and signal energy [52,53,54,55]. Figure 9a,b show the models for the two applications in this study, respectively, and Figure 9c,d show the 10% CBT/PVDF PENG demonstrated in this study for capturing the energy gained by dropping the ball at different heights (Video S2, Supplementary Materials) and the energy generated by the ball as it passes (Video S3, Supplementary Materials). Figure 9e,f show the corresponding voltage signals. Different magnitudes of voltage were obtained after dropping the ball at different heights, with the maximum voltage at 10 cm, 20 cm, 30 cm, 40 cm, 50 cm, 60 cm, 70 cm, 80 cm, 90 cm, and 100 cm being 4.68 V, 4.88 V, 6.32 V, 5.6 V, 4.8 V, 7.2 V, 6.88 V, 5.92 V, 9.4 V, and 12 V, respectively. As the drop height of the ball increases, the maximum voltage also shows an increasing trend. When a ball of the same mass is dropped from different heights, its velocity and kinetic energy vary, so as the height of the ball falling increases, the pressure generated when it hits the surface of the material also increases, generating a greater charge separation and potential difference, resulting in a higher voltage. As the small ball passes through the tube, it passes over the surface of the piezoelectric material fixed to the wall of the tube, which exerts a certain pressure on it and thus generates a corresponding voltage. The movement of a small ball is used to generate mechanical energy, which in turn generates electrical output. The principle can be applied to devices such as piezoelectric sensors and piezoelectric generators.

## 4. Conclusions

In summary, three-dimensional coral-like barium titanate was synthesized, PENGs based on CBT/PVDF composites were prepared, and the piezoelectric energy harvesting properties were investigated. The CBT/PVDF PENG (≈7 µm thick, 2 cm × 4 cm) generates 163 V and 16.7 µA at a compression force of 50 N. The three-dimensional coral-like structure of the CBT enhances connectivity and is essential for enhancing the performance of the PENG. Simulations demonstrate that the primary factor contributing to the enhanced performance is the high local stresses present in the CBT coral branch structure. The substantial local stresses are confirmed by XRD residual stress measurements in the CBT when the composite PENG is under external stress. The PENG showed good durability at 5000 cycles and lit up 50 commercial LEDs. The PENG generates a voltage of 4.68–12 V to capture the energy generated by the ball falling from different heights and a voltage of ≈0.55 V to capture the mechanical energy of the ball’s movement as it passes. This study suggests a CBT-based PENG with greatly improved energy harvesting capabilities for applications such as piezoelectric sensors and generators.

## Figures and Tables

**Table 1:** Comparison of the properties of PENG with different fillers and polymer composites with the present work.

Materials	Sample Size	Compression	V_oc_	I_sc_	Power@RL	Ref.
PZT/PVDF	2 × 0.9 cm^2^	8.5 Kpa	55 V	-	35 μWcm^−2^@10MΩ	[42]
PZT foam/PVDF	-	15% strain	84 V	1.6 μA	-	[37]
BT NPs/PVDF	1 × 1 cm^2^	10 Mpa	150 V	1500 nA	-	[18]
BT NPs-CNTs/PDMS	3 × 4 cm^2^	180° bending	3.2 V	0.35 μA	-	[43]
BaTiO_5_/PVDF	2 × 4 cm^2^	-	26 V	-	4.1 μWcm^−2^@22MΩ	[44]
(1-X)K_0.5_Na_0.5_NbO_3-x_BaTiO_3_/PVDF	3 × 3 cm^2^	0.4 N	100 V	22 nA	1.4 μWcm^−2^@100MΩ	[45]
BTFs/PDMS	2 × 4 cm^2^	50 N	220 V	19 μA	103.5 μWcm^−2^@10MΩ	[39]
CBT/PVDF	2 × 4 cm^2^	50 N	163 V	16.7 μA	52.5 μWcm^−2^@1MΩ	This work

## Data Availability

Data will be made available on request.

## Supplementary Materials

The following supporting information can be downloaded at: https://www.mdpi.com/article/10.3390/polym15153191/s1, Video S1: LEDs driven by piezoelectric nanogenerators; Video S2: Voltage output of a piezoelectric nanogenerator monitored by a ping-pong ball dropped from different heights; Video S3: Voltage output monitored as the sphere passes through the piezoelectric nanogenerator.
